# Congenital optic disc pits and optic disc pit maculopathy: a review

**DOI:** 10.3389/fopht.2023.1222979

**Published:** 2023-08-16

**Authors:** Ali Esmaeil, Ali Ali, Salman Almutairi, Khaled Alkandari, Raed Behbehani, Alaa Alali

**Affiliations:** ^1^ Department of Ophthalmology, Adan Hospital, Hadiya, Kuwait; ^2^ Faculty of Medicine, Kuwait University, Jabriya, Kuwait; ^3^ Vitreoretinal Service, Department of Ophthalmology, Ibn Sina Hospital, Kuwait, Kuwait; ^4^ Neuro-Ophthalmology Service, Department of Ophthalmology, Ibn Sina Hospital, Kuwait, Kuwait; ^5^ Pediatric Service, Department of Ophthalmology, Ibn Sina Hospital, Kuwait, Kuwait; ^6^ Vitreoretinal Service, Dasman Diabetes Institute, Kuwait, Kuwait

**Keywords:** optic disc pit, optic disc pit maculopathy, pars plana vitrectomy, retinoschisis, endolaser, gas tamponade

## Abstract

Optic disc pits are a rare but significant anomaly of the optic nerve head that can lead to visual impairment and associated complications. These pits are characterized by a small, oval-shaped depression in the disc, which can cause fluid accumulation and subsequent damage to the adjacent retina. Although the etiology and pathogenesis of optic disc pits are not fully understood, several theories have been proposed, including abnormal embryonic development and degenerative changes. Diagnosis is typically made through a comprehensive eye examination, including a dilated fundus exam and optical coherence tomography. Management options vary depending on the severity of the condition and associated complications, ranging from observation to surgical intervention.

## Introduction

Optic disc pit (ODP) is defined as the herniation of dysplastic retinal tissue into an excavation rich in collagen that often extends into the subarachnoid space through a defect in the lamina cribrosa. ODPs was first described by Weithe (1) and belong to a family of cavitary anomalies including optic disc coloboma, vacant optic disc, peripapillary staphyloma, and morning glory disk anomaly (2). This optic nerve head anomaly occurs at an estimated 2 in 10,000. ODP is predominantly unilateral (85%) and rarely bilateral (3).

OPDs are usually sporadic, with no underlying genetic etiology nor any gender predilection (4, 5). However, autosomal inheritance has been reported in some families (2, 6).

Optic disc pit maculopathy (ODP-M) is thought to arise from a net tractional gradient of pressure across the ODP (7, 8). A posterior vitreous detachment (PVD) creates tangential traction on the ODP, exacerbated by pressure build-up and the accumulation of subretinal fluid (8, 9). The pressure of the vitreous fluid is the only force counteracting detachment or retinoschisis. Thus, treatments in managing ODP-M aim to eliminate this pressure gradient by reducing traction on the ODP with either vitrectomy, applying pneumatic tamponade, sealing fluid passage from the pit to the fovea, or macular buckling.

This review aims to summarise the pathogenesis of ODP and ODP-M, delineate the natural history and examination findings of an ODP/ODP-M patient, and finally discuss the current methods used in the treatment of ODP-M.

## Pathophysiology

Optic pits develop as a result of improper closure of the optic fissure causing small depressions in the retinal margin usually in the inferio-temporal part of the optic nerve. These abnormalities lead fluid flowing into the pit, which in the initial stages leads to the formation of a schisis-like separation of the inner retinal layers (10). This will cause a centro-cecal scotoma, and later on, a dense central scotoma caused by a macular hole in the outer layer. Finally, the subretinal fluid will cause an outer retinal detachment (11).

**Figure 1:**
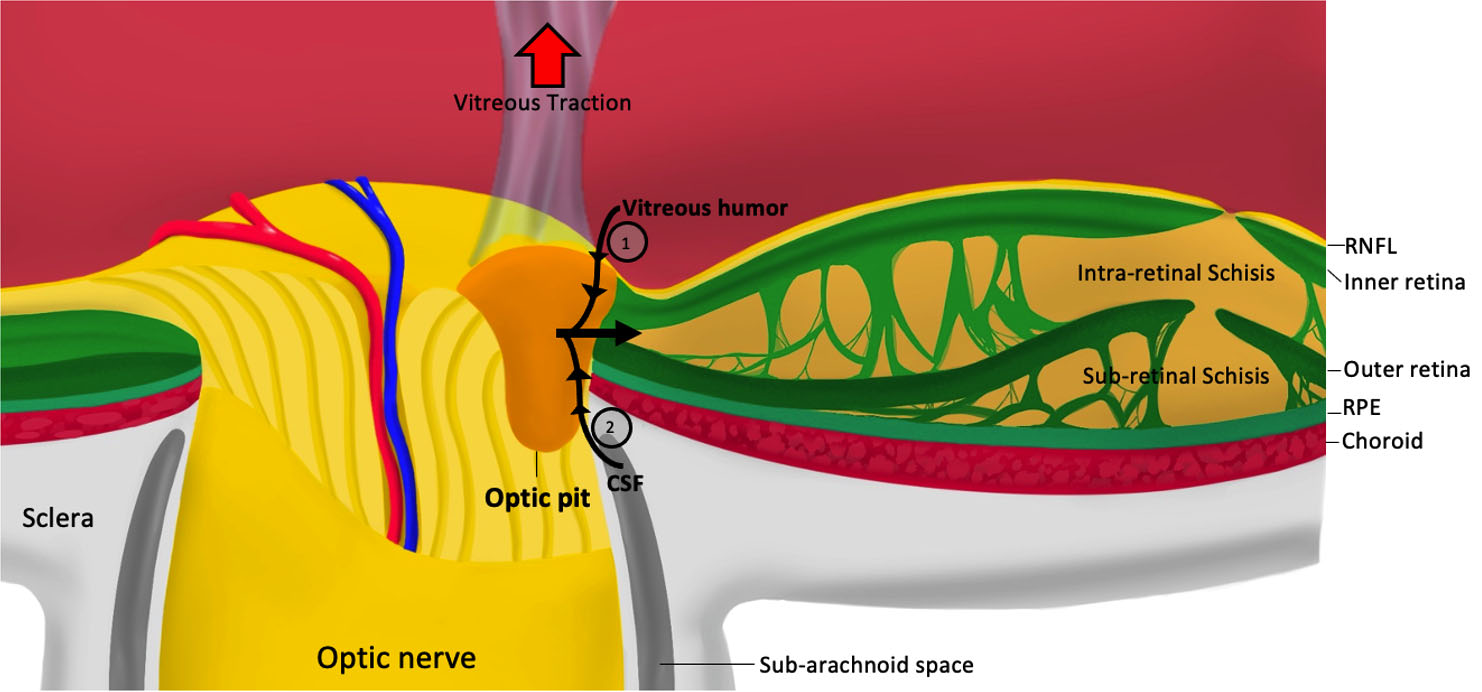
Pathophysiology of optic pit maculopathy. RNFL, retinal nerve fiber layer; RPE, retinal pigmented epithelium; CSF, cerebrospinal fluid; 1, pressure exerted by vitreous fluid; 2, Pressure exerted by cerebrospinal fluid.

The hallmark of ODP-M is intra- and sub-retinal fluid, however; the origin of the fluid and the exact mechanism of how the pit forms remain controversial. Proposed hypotheses suggest different sources of the fluid, including the vitreous (12–14), cerebrospinal fluid (15, 16), choroid, or blood vessels leaking from the pit’s base (17, 18). The mechanisms that have been put forward for ODP-M formation are vitreous traction and pressure gradients within the eye (19, 20) (**Figure 1**).

The role of vitreous traction in the pathogenesis of ODP is supported by its occurrence in with the age where progressive vitreous liquefication occurs (21). This is supported by the spontaneous resolution of maculopathy after complete detachment of the PVD and the resolution of vitreous traction (22). In addition, treatment with pars plana vitrectomy (PPV) by relieving the traction over the ODP also leads to the resolution of maculopathy in some cases (23). Furthermore, optical coherence tomography (OCT) has shown vitreous strands, and membrane development over the ODP before the development of maculopathy, or the disappearance of the membrane after maculopathy develops due to overlying vitreous traction (16, 24, 25). However, some OCT-based studies have found no evidence of vitreous traction over ODPs with the development of maculopathy, and others revealed recurrent ODP-M even following treatment with PPV (11, 26). Moreover, ODP-M can occur in adults without PVD and paediatric patients without vitreous changes (20). Lastly, a study using 3-dimentional SD-OCT scans revealed a three-fold connection between the vitreous cavity and subretinal and intraretinal spaces, and perineural spaces suggesting that the fluid has both a vitreous and cerebrospinal origin (27).

Other OCT studies and reports have shown a direct communication between the subarachnoid space and the subretinal space with the presence of an ODP defect (16). Case of intracranial silicon oil migration after retinal detachment repair have been reported (19). In morning glory disc anomaly, another congenital defect of the optic disc with a similar embryogenic cause, a subarachnoid to subretinal communication, was reported with metrizamide cisternography, although it was not demonstrated with intrathecal fluorescein (28, 29).

Intracranial pressure (ICP) can be transmitted into the ODP through the cerebrospinal fluid (CSF). Therefore, fluid can either flow from the vitreous humor to the subretinal space with low ICP or flow backward into the eye when ICP increases (20). The migration of vitreous substitutes after retinal detachment repair (silicone oil or gases) to subretinal, intraretinal, and intracranial regions with ODP patients may support this hypothesis (30).

Hypotheses proposing vitreous humor being the source of fluid responsible for ODP-M are based on studies of gas or silicone moving from the vitreous cavity into the subretinal space in eyes with cavitary abnormalities similar to ODP (31). Additionally, a histopathological study found mucopolysaccharides in the in the subretinal fluid, however; no glycosaminoglycans were detected, both being components of the vitreous (13). Furthermore, recent histological specimens examined by electron microscopy supports the presence of defects in a thin membrane overlying the optic pit, which allows access for the vitreous fluid to enter into the neurosensory retina. The study also demonstrated lamellar connections between the area of retinoschisis and the subretinal space, which provides a pathway for liquified vitreous into the subretinal space (14).

Finally, the choroid was considered as a potential source of the fluid due to peripapillary chorioretinal atrophy of Bruch’s membrane (17). However, subretinal fluid was not observed frequently in similar atrophic chorioretinal conditions. A recent case series using multimodal imaging showed leakage and small vessels around the pit, signifying the need for further appraisal of the vessels being a potential source of the fluid (18).

## Clinical examination and natural history

Patients with an ODP are usually asymptomatic and diagnosed incidentally on routine examination (32). Symptomatic patients, on the other hand, may present with a paracentral arcuate scotoma or an enlargement of the blind spot (33). The progression of ODP to optic disc maculopathy (ODP-M) occurs in 25 to 75% of patients (15, 34). These patients develop serous detachment or retinoschisis, or a combination of both (35). The visual acuity (VA) of these patients at presentation can vary between 20/25 to counting fingers (CF) (36, 37). Visual impairment depends on the extent of schisis or detachment and its duration. Furthermore, patients with ODP-M may report worsening of vision when bending over due to exaggerated tractional forces on the ODP created by gravity during such movement (38).

On fundoscopy, an ODP presents with a round or oval excavation/pit in proximity with the optic disc margin usually located in the inferior temporal quadrant of the disc but can be centrally located in 10 to 20% of cases (39).ODP’s are usually grey-white in colour and vary in size from one-eighth to one-quarter of the optic disc. Furthermore, the cavity occasionally is filled with fine tissue which is probably glial in origin. The optic disc has normal retinal vasculature, but in some patients, a vessel can disappear as it enters the pit and re-appears on the adjacent margin of the ODP. ODP-M manifests as intra-retinal fluid or neurosensory detachment, which can be seen on fundoscopy (40). It must be noted that the degree of macular elevation is not consistent with the size of the optic pit. In untreated and persistent ODP-M, macular and pigmentary changes can develop (41).

Visual field defects in ODP patients include an arcuate scotoma, enlarged blind spots, paracentral scotomas, central scotomas, nasal or temporal steps, altitudinal defects, or generalized depression (42). These defects can be attributed to the disruption of nerve fibers by the ODP (7).

## Optical coherence tomography in ODP and ODP-M

Retinoschisis is a prominent feature that shows on OCT images in various patterns, being either intra or sub-retinal in patients with ODP-M. They can also exhibit neurosensory detachment. Fluid tends to accumulate in the outer nuclear layer (94%), inner nuclear layer (81%), ganglion cell layer (44%), and sub-internal limiting membrane (13%) (11, 43, 44).. Frequently intraretinal fluid can be found in more than one retinal layer. Roy et al.,2013 reported retinal fluid in the outer nuclear layer (100%), and in 53% inner and outer retinal schisis with subretinal fluid (45). These findings suggest that fluid is most likely to enter the outer layers before traversing into either inner retinal or sub retinal layers.

Occult ODP is characterized as maculopathy without a clear disc pit in a clinical setting or on OCT. Reports have shown such cases with abnormal cavitations in the optic head’s stroma (46) and cavitations at the optic disc’s temporal edge (47). In the latter report, a possibility of a fine membrane overlying the optic disc was proposed to be protective of the schisis-like maculopathy. These findings imply that the fluid concentration in the stromal cavitations and ultimately in the subretinal and outer retinal spaces may be made possible by the existence of an imperfect membrane.

## Fluorescein angiography

ODP is stained in the later stages of fluorescein angiography (FA) after an early hypofluorescent period (48, 49). Late hyperfluorescence in 17 eyes with macular elevation was noted in a study by Theodossiadis et al.,1999 using FA and indocyanine green angiography (ICG), and it may be attributed to the dye leaking into the schisis cavity and subretinal fluid. Furthermore, recent studies reported leakage within the pit together with macular staining (18).

## Optical coherence tomography angiography

OCT Angiography (OCTA) in recent years contributed to the understanding of the vascular component of the disease while providing a better resolution and a valuable view of the microvasculature that surrounds the optic disc which cannot be shown on FA and ICG. In a study by Michalewska et al.,2020, OCTA yielded branch-like artifacts that resolved after vitrectomy, hyporeflective rings, and abnormal vasculature at the optic pit base (50). Another study using OCTA concluded with an association of decreased vascular density in some regions of the optic disc and a reduction in visual acuity with the presence of ODP (51).

## Management of ODP and ODP-M

Close observation of patients with optic pits is sensible as most patients remain asymptomatic until the 4^th^ decade of life (52). Furthermore, spontaneous resolution in those developing ODP-M has been described in several cases reports (22, 53, 54). In patients with a stable ODP or in less severe cases with mild ODP-M and minimal visual symptoms, diligent follow up may be appropriate.

In ODP-M patients with persistent retinal fluid or a poor visual acuity, surgical intervention may be justified (20). Although a variety of surgical techniques are available, pars plana vitrectomy (PPV) with variable adjuvant therapies has become the standard surgical intervention in the management of ODP-M. The aim of PPV is to induce a PVD and decrease the tractional forces on the optic pit in an aim to aid absorption of subretinal fluid. PPV can be combined with laser therapy, gas tamponade, inner retinal fenestration, autologous fibrin, or ILM peeling. PPV has shown a functional success rate of 50%-95% in the treatment of ODP-M and a reported visual acuity improvement of 50% observed in various case series and reports (38, 55).

A commonly used intervention is PPV combined with gas tamponade and endolaser with favourable outcomes achieved in multiple case series. Examples of such publications include an 11 patient case series treated with vitrectomy, gas tamponade, and endolaser, which achieved complete resolution of retinal fluid in 10 patients, with 82% of patients achieving 2 or more Snellen lines of vision (56). In another comparative retrospective case series, the use of PPV + gas tamponade + endolaser (group 1) was compared to the results of PPV alone (group 2) in the treatment of ODP-M. Both groups exhibited similar improvement in post-operative visual acuity; however, patients in group 1 had faster resolution of subretinal fluid compared to group 2 (57). A large multi-centre retrospective study of 46 patients with ODP-M examined the use of PPV with and without juxtapapillary laser photocoagulation. After a mean follow up of 44 months, patients in either group had similar functional and anatomical outcomes (58).

Inner retinal fenestration, which involves constructing a fluid passage into the vitreous cavity by means of a partial thickness retinotomy temporal to the optic disc, is done in combination with PPV. In an interventional case series carried by Ooto et al., 17 out of 18 eyes showed resolution of intra and sub-foveal fluid with macular detachment resolving approximately 6 months after surgery. Furthermore, significant improvement of BCVA (mean of 0.378 LogMAR) was achieved (59). Another 11 patient case series utilising fenestration achieved resolution of retinal fluid in all patients, with mean visual acuity improving from 20/80 at baseline to 20/32 at 12-months of follow-up (60).

**Figure 2:**
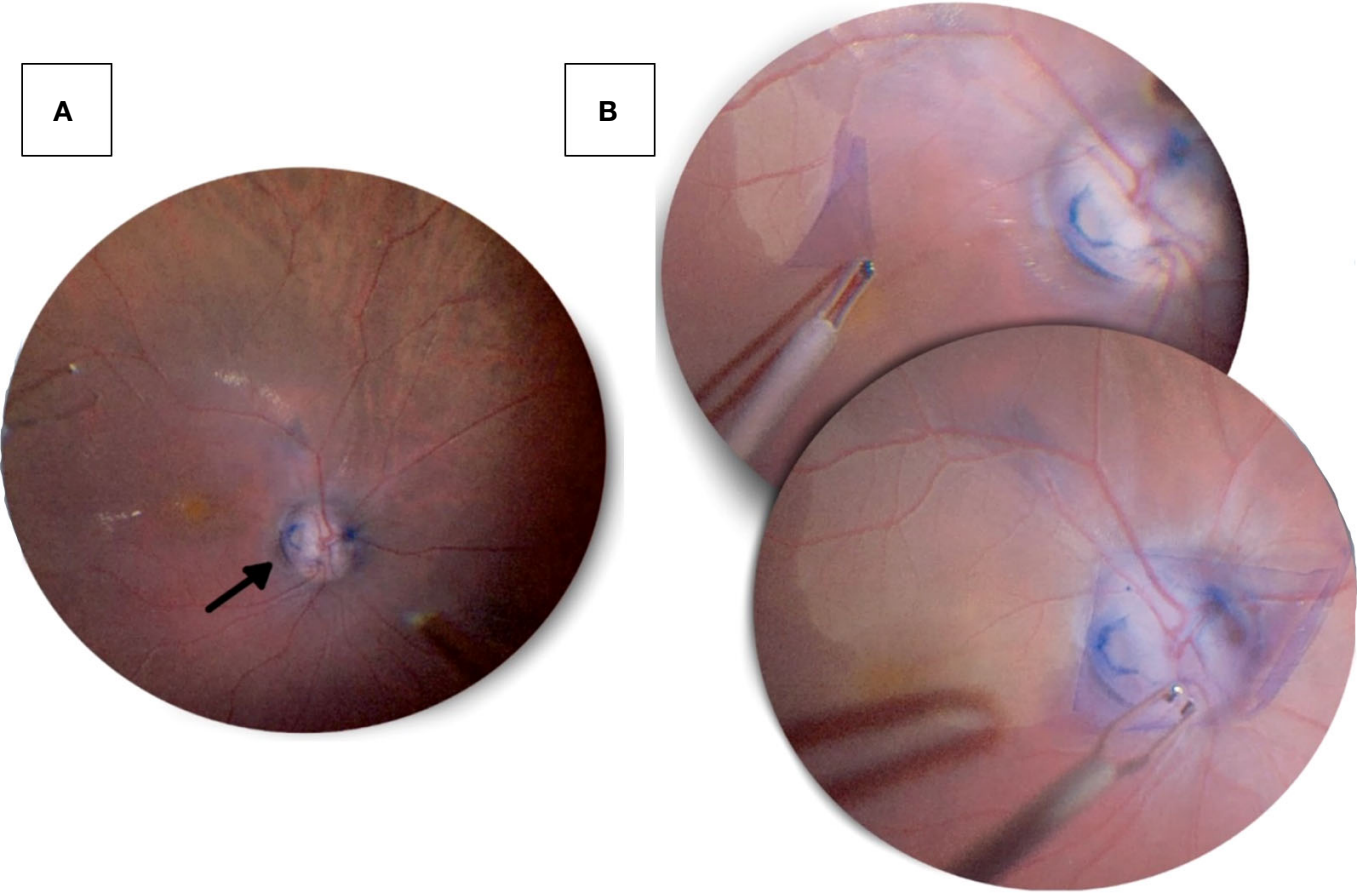
Pars plana vitrectomy with ILM peeling performed on a 47-year-old male patient’s right eye, who presented with progressive decrease in visual acuity. **(A)** An optic disc pit in the inferotemporal quadrant (black arrow) seen after a pars plana vitrectomy. **(B)** Following vitrectomy, the internal limiting membrane was peeled from the temporal aspect and placed over the optic disc with massaging to ensure prover coverage of the pit.

Another technique to treat ODP-M is ILM peeling, which can be particularly useful in cases of multilayer schisis (**Figure 2**) (55, 61). Currently, inverted ILM flaps have been used to enclose the optic disc and protect the fovea. This technique has shown promising outcomes in several case reports (62, 63). However, in a retrospective multi-centre case series, ILM peeling and endolaser treatment exhibited no advantage over stand-alone vitrectomy with tamponade (64).

The use of autologous platelet rich fibrin with PPV has been described in the literature. In one report, two patients with serous macular detachment persisting after PPV and ILM peeling, underwent autologous fibrin application over the optic disc pit followed by fluid-air-gas exchange. The retina remained attached for 27 months in the first case and 14 months in the second case (65). In another report, a patient had previously undergone vitrectomy and peripapillary laser but had recurrence of subretinal fluid and worsening visual acuity. PPV was repeated together with ILM peeling and autologous platelet rich plasma applied over the optic pit, with the addition of long-acting gas tamponade as a final step. When examined 8 months post-operatively, there was resolution of subretinal fluid and collapse of the connection between the optic pit and subretinal space, together with vision improvement from 20/100 to 20/50 (66). Thus, the incorporation of autologous fibrin in the treatment of refractory cases of ODP-M may be a viable option.

Macular buckling, in which a macular sponge is placed in the posterior part of the eye at the 6 to 12 o’clock meridian to generate the buckling effect needed beneath the macula, has been used for ODP-M Theodossiadis et al. (67). The buckle acts as a barrier to the entrance of fluid from the vitreous cavity or blocking the entrance of subarachnoid CSF into the retina. This technique has shown excellent long-term success with all 12 eyes examined over a period of 11 years exhibiting retained anatomical and functional results that they originally achieved 2 years postoperatively (68). The technique however is technically challenging and not frequently used at this time.

In-office treatments of ODP-M can be applied as an alternative to PPV especially in cases where operative intervention is not feasible. Intravitreal gas was shown to achieve retinal re-attachment in about 4 out of the 8 patients observed as part of a case series (69). Laser photocoagulation applied laterally to the optic disc creates chorio-retinal scarring will act as a barrier to fluid migration to the macula. On its own, laser photocoagulation showed sustained functional results with visual improvement in only a small number of patients (7). When intravitreal gas injection (C3F8) was combined with laser photocoagulation in a series of 8 patients, 75% showed complete resolution of intra-retinal and subretinal fluid with improvement in visual acuity (70). Another combination of intravitreal gas tamponade (C3F8) and laser photocoagulation applied 1 day after gas injection was examined in a 6 patient case series, achieving complete retinal reattachment in 4 patients with improvement in visual acuity in 5 patients (71). It must be noted however that laser photocoagulation may be accompanied with deleterious side effects that may affect central vision, which must be discussed with patients prior to treatment.

In an aim to reduce retinal fluid and help in the resolution of ODP-M a case report of a 27-year-old male patient who was refractory to PPV examined the use of oral spironolactone and 2% topical dorzolamide for 2 years and showed marked reduction in intraretinal fluid as well as macular schisis, with improvement of visual acuity from 20/50 to 20/30 (72). Another report exhibited a 56-year-old female who refused surgical intervention for ODP-M and was treated with topical dorzolamide. This patient had resolution of macular schisis and an improvement of visual acuity from 20/30 to 20/20 after 2 years of follow up (73). These unique reports give insight into the possible role of medical adjuvants to surgical intervention in ODP-M or an alternative when surgical intervention is not feasible.

## Conclusion

Optic disc pits are a relatively uncommon but potentially vision-threatening condition that affects the optic nerve head. The treatment options available include observation, laser therapy, and surgical intervention, with the choice of treatment dependent on the size, location, and number of pits as well as the degree of associated macular edema or subretinal fluid. The aim of treatment is to prevent progressive visual loss and to stabilize or improve vision. Regular monitoring is essential for early detection of changes, and appropriate intervention to preserve vision. Further research is required to improve our understanding of the pathogenesis of optic disc pits and to optimize their management.

## Author contributions

AE, AliA, SA, KA: Contributed to literature review and writing. RB, AlaA: Contributed to writing and final review of the paper. All authors contributed to the article and approved the submitted version.
